# The association between physical inactivity and obesity is modified by five domains of environmental quality in U.S. adults: A cross-sectional study

**DOI:** 10.1371/journal.pone.0203301

**Published:** 2018-08-30

**Authors:** Christine L. Gray, Lynne C. Messer, Kristen M. Rappazzo, Jyotsna S. Jagai, Shannon C. Grabich, Danelle T. Lobdell

**Affiliations:** 1 Department of Epidemiology, Gillings School of Global Public Health, University of North Carolina at Chapel Hill, Chapel Hill, North Carolina, United States of America; 2 Oak Ridge Institute for Science and Education at the U.S. Environmental Protection Agency, USEPA Human Studies Facility, Chapel Hill, North Carolina, United States of America; 3 School of Public Health, Oregon Health & Sciences University-Portland State University, Portland, Oregon, United States of America; 4 National Health and Environmental Effects Research Laboratory, U.S. Environmental Protection Agency, USEPA Human Studies Facility, Chapel Hill, North Carolina, United States of America; 5 Division of Environmental and Occupational Health Sciences, School of Public Health, University of Illinois Chicago, Chicago, Illinois, United States of America; University of Maiduguri College of Medical Sciences, NIGERIA

## Abstract

Physical inactivity is a primary contributor to the obesity epidemic, but may be promoted or hindered by environmental factors. To examine how cumulative environmental quality may modify the inactivity-obesity relationship, we conducted a cross-sectional study by linking county-level Behavioral Risk Factor Surveillance System data with the Environmental Quality Index (EQI), a composite measure of five environmental domains (air, water, land, built, sociodemographic) across all U.S. counties. We estimated the county-level association (N = 3,137 counties) between 2009 age-adjusted leisure-time physical inactivity (LTPIA) and 2010 age-adjusted obesity from BRFSS across EQI tertiles using multi-level linear regression, with a random intercept for state, adjusted for percent minority and rural-urban status. We modelled overall and sex-specific estimates, reporting prevalence differences (PD) and 95% confidence intervals (CI). In the overall population, the PD increased from best (PD = 0.341 (95% CI: 0.287, 0.396)) to worst (PD = 0.645 (95% CI: 0.599, 0.690)) EQI tertile. We observed similar trends in males from best (PD = 0.244 (95% CI: 0.194, 0.294)) to worst (PD = 0.601 (95% CI: 0.556, 0.647)) quality environments, and in females from best (PD = 0.446 (95% CI: 0.385, 0.507)) to worst (PD = 0.655 (95% CI: 0.607, 0.703)). We found that poor environmental quality exacerbates the LTPIA-obesity relationship. Efforts to improve obesity through LTPIA may benefit from considering this relationship.

## Introduction

Obesity affects more than one-third of adults in the United States (U.S.) [1, 2] and has been characterized as an ongoing epidemic [3]. Because obesity is a precursor to numerous poor outcomes, including functional impairment [4], diabetes [5], heart disease [6–8], cancer [9–11] and death [12], it has become a focal point for assessing the nation’s health [13]. Understanding its etiology and identifying prevention mechanisms are a public health priority [13, 14].

The causes of obesity are multifactorial. Individual-level contributors can arise from non-modifiable factors such as genetics [15] and family history [16], modifiable behaviors such as dietary intake [17–19] and physical activity [18–21], and socioeconomic circumstances such as poverty [22]. The food system—including processed foods, agricultural policies, and marketing—contributes to dietary intake and by extension, obesity [23–25]. Increasingly, the role of the ambient environment has been recognized as a critical element in the obesity epidemic [26]. Recent studies show numerous environmental associations with obesity, including environmental obesogens [27, 28] (e.g., bisphenol A (BPA), phthalates), food desserts [29], distance to parks and other built environment attributes [30], and air pollution [31]. Furthermore, these factors are all likely operating in tandem, creating a complex set of conditions in which obesity persists.

Physical inactivity is one of the primary contributors to the obesity epidemic in the U.S. [19] and is often targeted for intervention because it is modifiable at the individual level [32, 33]. One study examined the correlation of 20-year trends in leisure- time physical inactivity (LTPIA) and obesity and concluded that public health emphasis should be placed on physical inactivity, even over dietary intake [34]. However, the effects of physical inactivity on obesity may be impacted by environmental exposures. For example, increased air pollution, measured by particulate matter (PM_2.5_ and PM_10_) and ozone (O_3_), has been associated with increased odds of physical inactivity [35]. Features of the built environment, such as street connectivity and availability of parks and green space, have been explored as predictors of physical activity, though results have been mixed [36–38]. A systematic review of studies on the physical environment and physical activity indicated similarly inconsistent results regarding associations between environmental characteristics and physical activity [39]. Variations in physical activity resources have also been associated with sociodemographic factors: Jones et al (2015) found that lack of access parks and recreational facilities was associated with predominantly minority census tracts while low-income census tracts had as or greater availability of parks, but fewer recreational facilities [40].

The mixed results of these studies may stem from an inability to account for the multitude of environmental exposures occurring simultaneously. As the range of aforementioned studies suggests, there are many aspects of the environment that may modify (promote or hinder) the relationship between physical inactivity and obesity. Importantly, these environmental exposures occur simultaneously. While some studies control for specific environmental features such as census measures of percent poverty or median income [41–43], research to date has primarily been limited to examining single environmental exposures or single domains, such as sociodemographics. This is at least partly due to the challenge in empirically quantifying the broad range of cumulative exposures that affect human health [44]. However, the Environmental Quality Index (EQI) was recently developed by the U.S. Environmental Protection Agency (EPA) to address this limitation. The EQI is a publicly available, county-level measure of cumulative environmental exposures across five domains of the environment (air, water, land, built, and sociodemographic) [45]. The county-level EQI is the only environmental quality index of its nature in that it offers complete coverage of the United States, and incorporates variables from multiple domains that can impact the relationship between physical inactivity and obesity.

In this cross-sectional study, we use the EQI to examine how variations in county-level environmental quality impact the relationship between leisure-time physical inactivity (LTPIA) and obesity. examine how variations in county-level environmental quality effect measure modification (i.e., variation in the measure of association across the values of another variable, “modification”) by cumulative environmental quality in the association between county-level leisure-time physical inactivity (LTPIA) and county-level obesity. We additionally examine modification by each of the domain-specific indices that comprise the cumulative EQI. Because obesity rates differ by sex, in part due to complex socioeconomic dynamics [46], we examine associations in the overall population as well as by males and females separately.

## Materials and methods

### Data sources and variables

#### Behavioral Risk Factor Surveillance System (BRFSS)

The Behavioral Risk Factor Surveillance System (BRFSS) is a survey routinely conducted by states and reported to the Centers for Disease Control and Prevention. States annually sample a cross-section of the population through random digit dialing; respondents are asked a series of questions related to their health status and health behaviors, including, height, weight, and level of leisure time physical activity.

Leisure-time physical inactivity (LTPIA) is defined by answering “no” to the following question: “During the past month, other than your regular job, did you participate in any physical activities or exercises such as running, calisthenics, golf, gardening, or walking for exercise?”[47] County-level obesity is defined as percentage of the population weighing ≥ 30 kg/m^2^. From the self-reported BRFSS survey, body mass index (BMI) is calculated using height and weight; from that information, the percentage of obese individuals in the county is estimated [47].

For this study, we used BRFSS 2009 county-level, age-adjusted leisure-time physical inactivity (LTPIA) as our exposure and 2010 county-level, age-adjusted obesity as our outcome.[48] These years of data were chosen for two reasons: 1) sex-specific rates were available for 2009–2010 and 2) we were interested in keeping our outcome estimate temporally lagged from our exposure estimate. We used the age-adjusted estimates to account for differences in both LTPIA and obesity associated with age.

#### Environmental Quality Index (EQI)

The EQI is a county-level index constructed to reflect the relative quality of the cumulative environment across counties in all 50 states and Washington, DC from 2000–2005; data, including the original measured variables, and the accompanying technical report are publicly available [45]. Briefly, 187 data sources across five domains (air, water, land, built and sociodemographic environments) were examined for data quality, availability, and geographic coverage [49]. The 28 data sources retained after examination included empirical measurements of over 200 specific variables for counties across the U.S. The final index included 87 air domain variables (e.g., criteria and hazardous air pollutants), 80 water domain variables (e.g. chemical contaminants, drought, atmospheric deposition), 26 land domain variables (e.g., agriculture, pesticides, facilities), 14 built domain variables (e.g., highway/road safety, business environment), and 12 sociodemographic domain variables (e.g., percent low education, crime). To summarize this high-dimensional data, five domain-specific indices were generated by retaining the first component of a principal components analysis (PCA). These five domain indices were then used to create the overall EQI, again by retaining the first component of a PCA [44]. For this analysis, we used tertiles of the EQI and domain-specific indices to examine effect measure modification of the physical inactivity-obesity relationship by level of environmental quality.

#### U.S. census data

County race and ethnicity distributions may reflect social factors that affect where people live as well as county-level obesity. To account for this, we used data from the U.S. Census to create a variable representing the percentage minority in each county. Specifically, we used the percentage of residents who were anything other than non-Hispanic White, which included Black non-Hispanic, Hispanic, American Indian, Asian or Pacific Islander.

#### Rural-urban continuum codes

Rural-urban continuum codes (RUCC) are indicators of the degree of rurality (or urbanicity) for a particular county [50]. U.S. Department of Agriculture classifies counties into nine RUCC categories [51]; we have collapsed those into four categories, consistent with prior health studies [52–54]: metropolitan-urban (RUCC 1), non-metropolitan urban (RUCC 2), less urban (RUCC 3) and thinly populated (RUCC 4). In this analysis, we used indicator variables (RUCC 1 as the referent) to control for the vastly different environments that occur across the rural-urban spectrum.

### Statistical analyses

To estimate the association between age-adjusted LTPIA and age-adjusted obesity across tertiles of the EQI, we used multi-level linear regression models, with a random intercept for state, adjusted for county percent minority and RUCC. We stratified by tertiles of the EQI (rather than using interaction terms) because we were explicitly interested in how the association between LTPIA and obesity operates in different quality environments. Our estimates are reported as prevalence differences (PD) and 95% confidence intervals (CI).

We considered clustering by climate regions identified using Koppen Climate Regions [55], as in a prior analysis using the EQI [56], because environmental quality may vary by climate region and because climate region has been associated with obesity [57]. However, existing literature also suggests that state-level policies are particularly relevant to examination of physical activity and obesity trends [58–61]. We empirically assessed both state and climate region using intra-class correlation coefficients (ICC), a statistical measurement that estimates the extent of clustering, i.e., the extent to which units (here, counties) within a group (here, a state) are similar to each other with respect to the outcome (here, obesity). Clustering counties in a state had a higher ICC (0.58) compared to clustering by climate regions (ICC = 0.34), indicating that state accounted for more of the overall variance across counties. Therefore, we clustered our analyses by state.

In addition to the overall EQI, we examined effect measure modification by tertiles of domain-specific indices. In those analyses, we adjusted for the other environmental domains. All analyses were conducted in the overall population and separately by males and females using sex-specific rates.

All analyses were conducted in SAS version 9.4 [62].

## Results

### Descriptive analyses

Among the 3,137 counties in our analysis, most are metropolitan urbanized (n = 1089) or less urbanized (n = 1057) (Table 1). The mean percent minority was 19.4% (standard deviation (sd): 19.3). In 2010, the mean age-adjusted county-level prevalence of obesity for the overall population was 30.5% (sd: 4.3); it was slightly higher for males (31.3%, sd: 3.8) and slightly lower for females (29.6%, sd: 5.1). LTPIA in 2009 was about 2.5% lower in males than females, and averaged 26.9% (sd: 4.9) for the overall population.

**Table 1:** Characteristics of counties in the United States, overall and by tertile of the Environmental Quality Index (EQI).

	Overall		EQI Tertile 1: Best Quality		EQI Tertile 2: Middle Quality			EQI Tertile 3: Worst Quality	
	(N = 3,137 counties)		(N = 1,043 counties)		(N = 1,047 counties)			(N = 1,047 counties)	
Characteristic	mean (sd)		mean (sd)		mean (sd)			mean (sd)	
2009 Age-Adjusted LTPIA									
Overall	26.9 (4.9)		29.3 (4.4)		27.3 (4.4)			24.1 (4.6)	
Males	25.6 (4.7)		27.8 (4.1)		26.0 (4.2)			22.8 (4.4)	
Females	28.1 (5.5)		30.7 (5.1)		28.5 (4.9)			25.2 (4.9)	
2010 Age-Adjusted Obesity									
Overall	30.5 (4.3)		32.2 (4.2)		30.8 (3.7)			28.4 (4.2)	
Males	31.3 (3.8)		32.7 (3.5)		31.6 (3.2)			29.6 (4.1)	
Females	29.6 (5.1)		31.5 (5.3)		29.9 (4.4)			27.2 (4.5)	
Percent Minority	19.4 (19.3)		26.0 (23.8)		17.3 (17.0)			14.9 (13.7)	
	N (%)		N (%)		N (%)			N (%)	
Rural-Urban Category									
Metropolitan urbanized	1089 (34.7)		146 (14.0)		299 (28.6)			644 (61.5)	
Non-metro urbanized	323 (10.3)		37 (3.5)		123 (11.8)			163 (15.6)	
Less urbanized	1057 (33.7)		378 (36.1)		460 (43.9)			221 (21.1)	
Thinly populated	668 (21.3)		486 (46.4)		165 (15.8)			19 (1.8)	

CI: confidence interval; EQI: Environmental Quality Index; PD: prevalence difference; SD: standard deviation

### Associations between LTPIA and obesity

Prior to stratification by environmental quality, the association between LTPIA and obesity, adjusted for RUCC and percent minority, was PD = 0.512 (95% CI: 0.483, 0.540) overall, 0.443 (95% CI: 0.416, 0.470) for males, and 0.565 (95% CI: 0.534, 0.595) for females.

### Modification by overall environmental quality

**Fig 1:**
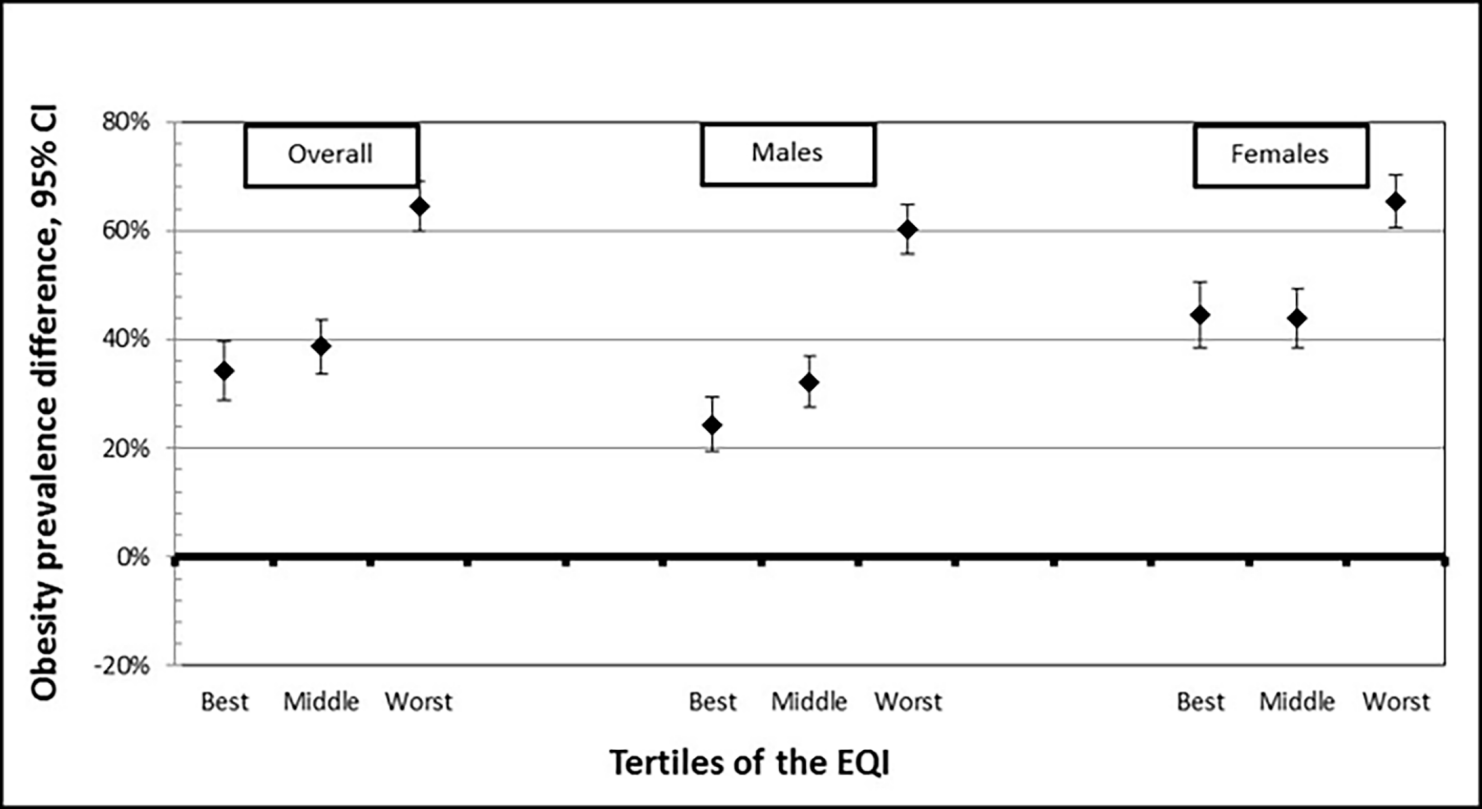
Association between leisure-time physical inactivity (LTPIA) and obesity by Environmental Quality Index (EQI) tertile, overall and by sex. (N = 3,137 counties).

Stratified results indicate effect measure modification by overall environmental quality (Fig 1; S1 Table). For the overall population, the PD increases 14% from the best (PD = 0.341 (95% CI: 0.287, 0.396)) to the middle (PD = 0.388 (95% CI: 0.338, 0.437)) tertile of environmental quality, and increases another 66% from middle to worst (PD = 0.645 (95% CI: 0.599, 0.690)) tertile of environmental quality. We observed a similar trend in males. Among females, we saw a very slight decline in PD from best (PD = 0.446 (95% CI: 0.385, 0.507)) to middle (PD = 0.438 (95% CI: 0.384, 0.492)) tertile, but a large increase in PD from both best and middle to the worst (PD = 0.655 (95% CI: 0.607, 0.703)) tertile of environmental quality. Across all tertiles, the association between LTPIA and obesity was larger in females than in males.

### Modification by air quality

**Fig 2:**
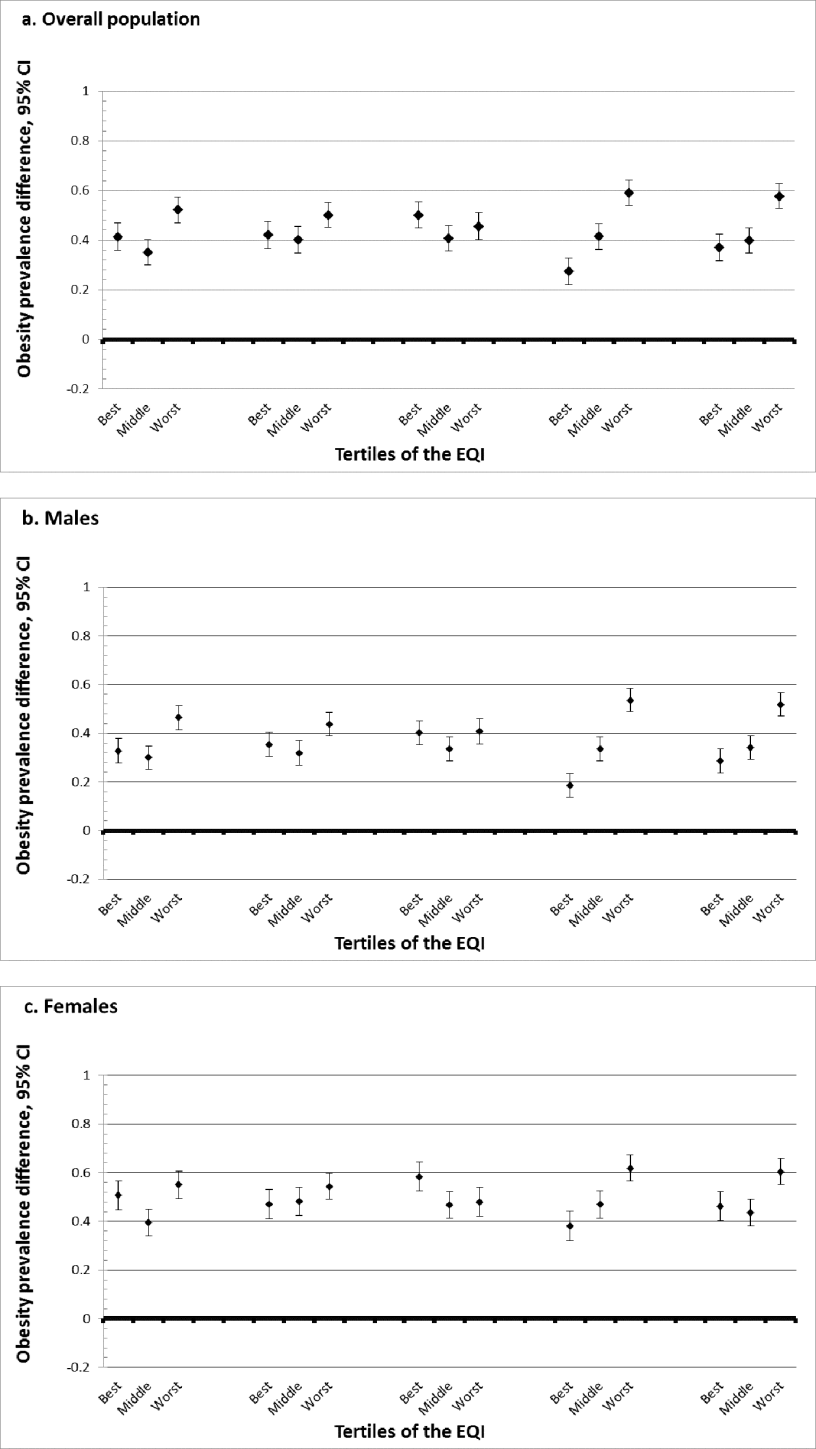
a-c. Association between leisure-time physical inactivity (LTPIA) and obesity by domain-specific Environmental Quality Index (EQI) tertile, overall and by sex. (N = 3,137 counties).

We further examined effect measure modification by domain-specific indices (Fig 2A–2C; S2 Table). In the air domain, the middle tertile quality environment had the smallest association overall (PD = 0.351 (95% CI: 0.301, 0.401)), as well as for both males (PD = 0.300 (95% CI: 0.253, 0.347)) and females (PD = 0.395 (95% CI: 0.340, 0.451)). The worst quality environment in the air domain had the largest association in all three populations: overall (PD = 0.522 (95% CI: 0.470, 0.575)), males (PD = 0.465 (95% CI: 0.414, 0.516)), and females (PD = 0.550 (95% CI: 0.493, 0.607)).

### Modification by water quality

In the water domain, estimates in the best and middle tertiles were similar in the overall population, in males, and in females(Fig 2A–2C; S2 Table). As with the air domain, the largest prevalence differences were in the worst quality environments: overall (PD = 0.501 (95% CI: 0.451, 0.551)), males (PD = 0.438 (95% CI: 0.390,0.485)), and females (PD = 0.550 (95% CI: 0.490,0.597)).

### Modification by land quality

The land domain was the only domain in which the largest association was not in the tertile with the worst quality environment; the modification was also not as pronounced in the land domain as in other domains (Fig 2A–2C; S2 Table). In the overall population, the association in the best tertile (PD = 0.501 (95% CI: 0.449, 0.554)) was somewhat higher than the worst (PD = 0.457 (95% CI: 0.401, 0.512)). For males, the best (PD = 0.401 (95% CI: 0.352, 0.450)) and worst (PD = 0.408 (95% CI: 0.355, 0.461)) were about the same. For females, the best quality tertile (PD = 0.584 (95% CI: 0.525, 0.642)) was larger than the worst (PD = 0.480 (95% CI: 0.420, 0.540)).

### Modification by built environment quality

Modification by environmental quality was most pronounced in the built environment domain. In the overall, male and female populations, the prevalence difference substantially and monotonically increases from best to worst quality environment (Fig 2A–2C; S2 Table). For the overall population, the association increases from PD = 0.276 (95% CI: 0.222, 0.330) in the best tertile to PD = 0.591(0.541, 0.641) in the worst. Among males, the association increases from PD = 0.184 (95% CI: 0.135, 0.233) in the best tertile to PD = 0.536 (95% CI: 0.487, 0.585) in the worst tertile. In females, the association increases from PD = 0.381 (95% CI: 0.319, 0.442) in the best to PD = 0.618 (95% CI: 0.565, 0.671) in the worst.

### Modification by sociodemographic environment quality

While the modification was not as pronounced as the built domain, the sociodemographic domain similarly indicated much larger associations in the worst tertile compared to the middle and best tertiles (Fig 2A–2C; S2 Table). The prevalence differences in the worst tertiles were PD = 0.578 (95% CI: 0.528, 0.627) for the overall population, PD = 0.518 (95% CI: 0.470, 0.566) for males, and PD = 0.604 (95% CI: 0.551, 0.657) for females.

## Discussion

We found that the association between LTPIA and obesity is modified by environmental quality, defined by five domains: air, water, land, built and sociodemographic environments. Specifically, in worst cumulative quality environments, the obesity prevalence associated with LTPIA in the overall population is much higher in the worst quality environments (PD: 0.645 (95% CI: 0.599, 0.690) compared to best quality environments (PD = 0.341 (95% CI: 0.287, 0.396)), and this finding holds among both males and females.

In nearly every domain, the prevalence difference in the worst quality tertile was larger than the middle and best quality tertiles. These findings are consistent with existing studies that have identified single-exposure associations with obesity in individual domains. Air pollutants and environmental obesogens, chemicals that disrupt lipid metabolism and can accumulate in the air, water, and land, have been associated with obesity in prior studies [27, 28, 42]. The built and sociodemographic domains in particular had much higher prevalence differences in the worst tertile (built PD: 0.591 (95% CI: 0.541, 0.641); sociodemographic PD: 0.578 (95% CI: 0.528, 0.627)) compared to the best (built PD: 0.276 (95% CI: 0.222, 0.330); sociodemographic PD: 0.371 (95% CI: 0.318, 0.424)), which is also consistent with prior findings that increased distance to parks [30] and quality food [29], as well as low socioeconomic status [46, 63] are associated with obesity. We note that in many cases, there is minimal difference between the best and middle quality tertiles. It may be that there is a threshold effect wherein the worst quality tertile is particularly harmful. The exception in our findings was the land domain, where there was less evidence of modification in general and where the highest magnitude associations were in counties in the best quality tertile. The land domain represents environmental aspects that may not have as much influence on LTPIA. Specifically, one aspect of the land domain reflects presence of pesticides used in agriculture; it is possible that those living in counties with the worst land quality (potentially the most agricultural pesticides) are more engaged in physically active labor and less influenced by *leisure time* physical activity in general.

One implication is that our findings may help explain some of the variation and inconsistent results observed in prior studies of physical inactivity and obesity [36–39]. For example, while street connectivity may be a positive attribute that facilitates walkability, it may coexist with negative attributes such as higher crime rates, increased air pollution, or reduced green space that limit outdoor activity. By using the cumulative EQI, we were able to account for numerous co-occurring exposures across multiple environmental domains. Breaking the EQI down into domain-specific indices allowed us to examine which domains may be important drivers of the association between LTPIA and obesity while still controlling for each of the other domains. Both the built and sociodemographic domains had the most pronounced differences moving from the best to worst tertiles of environmental quality, suggesting these environments may be particularly important intervention targets to improve public health as it relates to LTPIA and obesity.

The association between LTPIA and obesity is larger in females than in males in all tertiles of environmental quality, in the cumulative EQI and in the domain-specific indices. However, the difference between the best and worst tertiles was more pronounced in males than females; males seemed to experience greater benefit in the best environments as compared to the worst environments. The EQI accounts for crime rates, which are commonly thought to be a greater concern among women, and have been associated with reduced physical activity for women [64]. It is possible that while the EQI accounts for many ambient exposures, including crime, across multiple domains, females may experience some unmeasured element of the environment more acutely than males, limiting our ability to observe the same level of distinction between the worst and best quality environments. For example, at least one study in the United Kingdom showed that perceived quality of green space is more important than quantity [65]. Females may be particularly affected by such factors given their conditioning to be more constantly vigilant of potential threats in their environment.

This study has some limitations. BRFSS measures of LTPIA and obesity are reliant on self-report data obtained from random-digit dialing. In particular, weight, which can be a sensitive topic, may be under-reported. However, in comparison to other national surveys, including one with anthropometric measurement, national BRFSS estimates were found to be consistent with other surveys, supporting the validity of BRFSS estimates [66, 67]. Random-digit dialing may result in differential sampling that can bias results, particularly since the years in this analysis pre-date sampling of cell phones. However, the National Health Interview Survey, with which BRFSS estimates are consistent, uses a representative sampling of addresses of households and non-institutional group quarters (e.g., college dormitories). Another limitation is that the EQI reflects county-level environmental quality from 2000–2005, a few years before the BRFSS estimates for LTPIA and obesity. However, the variables in the EQI are generally stable over time and we do not expect that the relative ranking of counties into best and worst environmental quality tertiles would shift meaningfully in that short of a time lag. Finally, use of county-level measures prevents individual-level inference. However, the use of county-level measures enable assessment that can be useful to public health practitioners hoping to better understand and advocate for county-wide improvements in environmental quality as part of their obesity programs.

We also note several strengths to this study. We linked multiple public data sources to conduct this study of counties across the entire U.S., providing a more generalizable analysis than studies limited to specific geographic areas. By controlling for rural-urban status, we were able account for environmental variability by degree of rurality while including populations in both rural and urban areas to represent the entire U.S. Using 2009 LTPIA estimates and 2010 obesity estimates allowed us to ensure the outcome was temporally subsequent to the exposure, preventing the potential for reverse causation. Finally, the EQI provided a novel and comprehensive measure of environmental quality, leveraging information from numerous variables across five domains of the environment and incorporating variability of environments across the U.S. In doing so, it enabled a better understanding how the LTPIA–obesity relationship is facilitated by the many simultaneous exposures affecting human health.

## Conclusion

Our study showed that the county-level association between physical inactivity and obesity is significantly exacerbated by poor cumulative environmental quality. The EQI, which incorporates five domains of environmental quality (air, water, land, built and sociodemographic), provided a metric that addressed a gap in quantitative approaches to controlling myriad aspects of the environment. Future research, as well as programs aimed at improving population-level obesity through physical activity, may benefit from considering the role of cumulative environmental quality in the obesity epidemic.

## Supporting information

S1 Table Associations between LTPIA and obesity for each EQI tertile, for the overall population, males, and females. (DOCX)

S2 Table Associations between LTPIA and obesity for each domain-specific EQI tertile, for the overall population, males, and females. (DOCX)
