# Supplementary material for: The association between physical inactivity and obesity is modified by five domains of environmental quality in U.S. adults: A cross-sectional study
Source: PLoS One. 2018 Aug 30;13(8):e0203301. doi: 10.1371/journal.pone.0203301 (PMC6117021; doi:10.1371/journal.pone.0203301)
Supplement: S1 Table — (DOCX) [file pone.0203301.s001.docx]

| Supplemental Table 1. Associations Between LTPIA and Obesity for Each EQI Tertile, for the Overall Population, Males, and Females | | | |
| --- | --- | --- | --- |
|  |  |  |  |
| EQI Tertile | Overall population | Males | Females |
|  | PD (95% CI) | PD (95% CI) | PD (95% CI) |
| Best | 0.341 (0.287,0.396) | 0.244 (0.194,0.294) | 0.446 (0.385,0.507) |
| Middle | 0.388 (0.338,0.437) | 0.322 (0.275,0.369) | 0.438 (0.384,0.492) |
| Worst | 0.645 (0.599,0.690) | 0.601 (0.556,0.647) | 0.655 (0.607,0.703) |

CI: confidence interval; EQI: Environmental Quality Index; PD: prevalence difference
