# Supplementary material for: The association between physical inactivity and obesity is modified by five domains of environmental quality in U.S. adults: A cross-sectional study
Source: PLoS One. 2018 Aug 30;13(8):e0203301. doi: 10.1371/journal.pone.0203301 (PMC6117021; doi:10.1371/journal.pone.0203301)
Supplement: S2 Table — (DOCX) [file pone.0203301.s002.docx]

| Supplemental Table 2. Associations Between LTPIA and Obesity for Each Domain-Specific EQI Tertile, for the Overall Population, Males, and Females | | | | | |
| --- | --- | --- | --- | --- | --- |
|  |  |  |  |  |  |
| Domain-Specific EQI Tertiles | Overall population |  | Males |  | Females |
|  | PD (95% CI) |  | PD (95% CI) |  | PD (95% CI) |
| Air EQI |  |  |  |  |  |
| Best | 0.414 (0.359,0.469) |  | 0.328 (0.276,0.380) |  | 0.507 (0.447,0.566) |
| Middle | 0.351 (0.301,0.401) |  | 0.300 (0.253,0.347) |  | 0.395 (0.340,0.451) |
| Worst | 0.522 (0.470,0.575) |  | 0.465 (0.414,0.516) |  | 0.550 (0.493,0.607) |
| Water EQI |  |  |  |  |  |
| Best | 0.420 (0.365,0.475) |  | 0.354 (0.303,0.405) |  | 0.470 (0.409,0.531) |
| Middle | 0.402 (0.348,0.457) |  | 0.319 (0.268,0.370) |  | 0.481 (0.423,0.540) |
| Worst | 0.501 (0.451,0.551) |  | 0.438 (0.390,0.485) |  | 0.544 (0.490,0.597) |
| Land EQI |  |  |  |  |  |
| Best | 0.501 (0.449,0.554) |  | 0.401 (0.352,0.450) |  | 0.584 (0.525,0.642) |
| Middle | 0.408 (0.357,0.460) |  | 0.336 (0.287,0.385) |  | 0.468 (0.413,0.522) |
| Worst | 0.457 (0.401,0.512) |  | 0.408 (0.355,0.461) |  | 0.480 (0.420,0.540) |
| Built EQI |  |  |  |  |  |
| Best | 0.276 (0.222,0.330) |  | 0.184 (0.135,0.233) |  | 0.381 (0.319,0.442) |
| Middle | 0.415 (0.363,0.467) |  | 0.337 (0.287,0.386) |  | 0.469 (0.412,0.526) |
| Worst | 0.591 (0.541,0.641) |  | 0.536 (0.487,0.585) |  | 0.618 (0.565,0.671) |
| Sociodemographic EQI |  |  |  |  |  |
| Best | 0.371 (0.318,0.424) |  | 0.287 (0.238,0.336) |  | 0.463 (0.404,0.521) |
| Middle | 0.399 (0.347,0.450) |  | 0.341 (0.292,0.390) |  | 0.436 (0.381,0.492) |
| Worst | 0.578 (0.528,0.627) |  | 0.518 (0.470,0.566) |  | 0.604 (0.551,0.657) |

CI: confidence interval; EQI: Environmental Quality Index; PD: prevalence difference
